# Current and further outlook on the protective potential of *Antrodia camphorata* against neurological disorders

**DOI:** 10.3389/fphar.2024.1372110

**Published:** 2024-04-17

**Authors:** Weiling Li, Pin Wan, Jialu Qiao, Yuchen Liu, Qian Peng, Zehua Zhang, Xiji Shu, Yiyuan Xia, Binlian Sun

**Affiliations:** Hubei Key Laboratory of Cognitive and Affective Disorders, Wuhan Institute of Biomedical Sciences, School of Medicine, Jianghan University, Wuhan, China

**Keywords:** *Antrodia camphorata*, neuroprotective activities, CNS disease, secondary metabolite, gut-microbiome-brain axis

## Abstract

Prevalent neurological disorders such as Alzheimer’s disease, Parkinson’s disease, and stroke are increasingly becoming a global burden as society ages. It is well-known that degeneration and loss of neurons are the fundamental underlying processes, but there are still no effective therapies for these neurological diseases. In recent years, plenty of studies have focused on the pharmacology and feasibility of natural products as new strategies for the development of drugs that target neurological disorders. *Antrodia camphorata* has become one of the most promising candidates, and the crude extracts and some active metabolites of it have been reported to play various pharmacological activities to alleviate neurological symptoms at cellular and molecular levels. This review highlights the current evidence of *Antrodia camphorata* against neurological disorders, including safety evaluation, metabolism, blood-brain barrier penetration, neuroprotective activities, and the potential on regulating the gut-microbiome-brain axis. Furthermore, potential strategies to resolve problematic issues identified in previous studies are also discussed. We aim to provide an overview for the ongoing development and utilization of *Antrodia camphorata* in cerebral neuropathology.

## 1 Introduction

*Antrodia camphorata* (M. Zang & C.H. Su) Sheng H. Wu, Ryvarden & T.T. Chang (AC) also called *Antrodia cinnamomea* or *Taiwanofungus camphoratus* or *Ganoderma camphoratum*, locally known as Niu-Chang-Chih in (Su et al., 2022), is a valuable edible mushroom, with a large potential for biological and medicinal health benefits including anti-cancer, anti-inflammatory, anti-oxidative, hepatoprotective, and neuroprotective properties (Zhang et al., 2022). Naturally, the growth of AC is extremely slow and parasitic on the inner wall of a unique and native tree in Taiwan called *Cinnamomum kanehirai* Hayata on the mountain ranges between 450 to 1200 m higher (Li et al., 2022; Su et al., 2022). AC was first identified in 1990, and was recognized and used as highly beneficial Chinese folk medicine (Chen et al., 2023). As a fungus, AC belongs to the phylum Basidiomycota, the Fomitopsidaceae family, and the *Antrodia* genus (Li et al., 2023). The appearance of fruiting bodies is generally red-orange, but in certain regions of Taiwan, rarely yellow and white variants also occur. Metabolomic Profiling indicated that red AC possesses relatively higher contents of triterpenoids and diverse metabolites than yellow AC and white AC (Su et al., 2023).

Ever since the spread of AC to the mainland, products like camphor mushroom drop pills and camphor mushroom oral liquid have become popular in the form of healthcare products (Cheng et al., 2005). Wild-grown AC is rare and valuable, but demands have increased in recent years. Therefore, research has been carried out on artificial cultivation including solid state (cutting wood, agar plate medium) culture and liquid culture (submerged fermentation) (Zhang et al., 2019). Although cultured fungus may possess bioactivities similar to those of the naturally occurring fungus, there are several differences in the constituents of ingredients and the content of bioactive metabolites (Du et al., 2012; Tung et al., 2014).

Currently, more than 200 metabolites have been extracted and identified from AC. Many investigations have revealed their pharmacological activities and mechanisms, and some of them are generally recognized by the U.S. Food and Drug Administration (FDA) as potential drugs for clinical trials (Angamuthu et al., 2019). Each stage of the fungal life cycle creates metabolites usually differently. Mycelium, an exponential phase of AC, and polysaccharide is usually produced in this stage (Zhang et al., 2018). It is not easy to artificially cultivate fruiting bodies. The chemical metabolites of the fruiting body are different from the mycelium, and in general, the metabolites of the mycelium are also found in the fruiting body; more secondary metabolites are produced during this mature phase (Chang et al., 2006).

The physiologically active substances in AC can be mainly divided into triterpenoids, polysaccharides, derivatives of ubiquinone, and derivatives of maleic acid and succinic acid (Liu et al., 2023). Triterpenoids are one of the main metabolites of AC, and the content of triterpenoids is approximately 63% in the fruiting body (Geethangili and Tzeng, 2011). Antcins, a typical class of triterpenoids, have high medicinal activity (Kuang et al., 2021). AC polysaccharides are mainly composed of a variety of monosaccharides linked by glycosidic bonds (Lee et al., 2002) and exhibit excellent biological activity of anticancer and anti-inflammatory properties (Yang et al., 2022). Ubiquinones are a class of lipophilic quinones and antroquinonol, probably the most valuable derivatives of ubiquinone in AC, has strong biological activity (Zhang et al., 2017). Maleic acid and succinic acid derivatives also are characteristic active metabolites of AC, mainly antrodin and antrocinnamomin, which are mainly found in the mycelium stage (Nakamura et al., 2004).

Neurological diseases such as Alzheimer’s disease (AD), Parkinson’s disease (PD), and stroke are becoming serious public health issues, and currently there is still no effective cure or prevention strategies. AD is the most common cause of dementia in older individuals with the pathological hallmarks of amyloid plaques composed of amyloid-β (Aβ) and neurofibrillary tangles consist of phosphorylated tau protein (Kalampokini et al., 2019; Marino et al., 2020). Currently, approved drugs to treat AD are mainly effective in improving the symptoms (Alzheimer’s Association, 2023; Yan et al., 2020). PD is the second-most common neurodegenerative disease, the pathological characteristics include the loss of dopaminergic neurons in the substantia nigra and the increase of Lewy body, which is caused by aggregation of α-synuclein in neurons (Beitz, 2014; Breijyeh et al., 2020). Current treatment of PD is limited to symptomatic relief (So et al., 2024). Stroke is one of the primary causes of disability and death worldwide, and ischemic stroke is the most common type (Zhao et al., 2022). Intravascular thrombosis is the primary pathogenic cause of ischemic stroke to result in brain damage, including cerebral tissue lesions and deficiencies in the neurons (Das et al., 2023). Therefore, preventative and improve of neural injuries strategies caused by stroke are of great clinical value.

**FIGURE 1:**
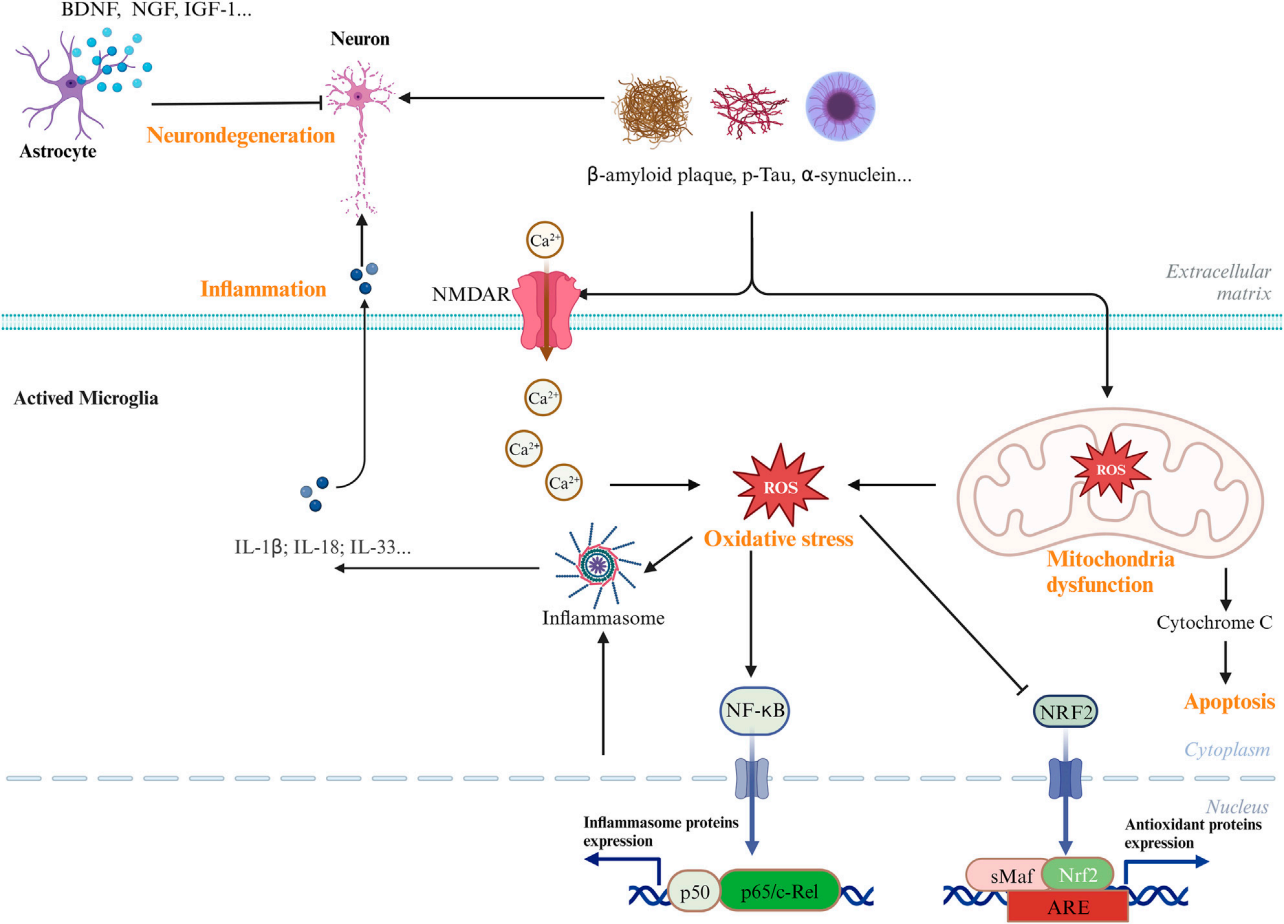
Common and major pathways in the CNS diseases. Biological processes, including oxidative stress, neuroinflammatory, and mitochondrial dysfunctions, have been involved in the development and pathogenesis of CNS diseases.

Although various clinical manifestations are present in neurological disorders, common signaling pathways are shared by AD, PD, and ischemic stroke, including neuronal cell death, inflammation, and oxidative stress (Sun et al., 2022). Figure 1 shows the common signaling pathways in neurological disorders. Hence, research on the inhibition of the common pathway will be useful for the development of new drugs against all neurological disorders. AC has been reported to have excellent neuroprotective effects. In this focused review, we summarize the most recent findings on the neuroprotective properties of the extracts and bioactive metabolites of AC.

## 2 Safety evaluation of AC

Traditionally, AC has been used as a health food, and the historical use of this mushroom as a food supplement supports its safety. Previous studies on genotoxicity, teratotoxicity, and oral toxicity have showed the no-observed-adverse-effect-level (NOAEL) of AC in Sprague–Dawley (SD) rats (Lin et al., 2015). A recent study showed that fruiting body powders of dish-cultured AC did not cause mortality and clinical symptoms of toxicity (Liu et al., 2022). The freeze-dried mycelium of AC can be considered as a novel food by the European Commission, and the European Food Safety Authority (EFSA) Panel on Nutrition, Novel Foods, and Food Allergens has reported no adverse effects at the proposed use level (Turck et al., 2022). In addition to showing an acceptable safety and tolerability profile at doses of up to 2,988 mg/day with no appreciable side effects, a phase I clinical study in healthy adults also raised the possibility that LEAC-102 (a novel botanical drug extracted from AC, major compounds are antcin B, antcin K, and antcin H) may have novel immunomodulatory activities (Liao et al., 2023). Taken together, these findings indicate that crude extracts of AC and its products have no adverse effects on humans within a certain dose range.

The toxicology of metabolites from AC also has been studied. To assess the safety of antroquinonol, a phase I clinic trial was carried out using 50–600 mg daily for 1 month in patients with metastatic non-small-cell lung cancer. Antroquinonol exhibited a mild toxicity profile at all dose levels. Of the five patients with high does at 600 mg, three were evaluable for treatment response, and two achieved stable disease, which was generally considered safe and tolerable without dose-limiting toxicities. Then, the recommended dose for phase II clinic trial is at least 600 mg daily for non-small-cell lung cancer (Lee et al., 2015).

β-glucan (∼65% pure) from AC was analyzed in the subchronic toxicity and mutagenicity study in CD (SD) IGS rats (∼6 weeks old, 12/sex/group), and a daily dose of 2 g/kg for 3 months of oral treatment did not show any adverse effects and genotoxicity (Chen et al., 2018). Therefore, AC and some of its bioactive metabolites showed no obvious toxicity within a reasonable dose range.

## 3 Metabolism and blood-brain barrier penetration of AC

As a dietary supplement and adjuvant therapeutic agent, AC is widely used, and the biological activities of different extracts of AC have been thoroughly investigated. Understanding the biological effects and safety of botanical drugs highly depends on preclinical research regarding the metabolism and pharmacokinetics.

To investigate the metabolism of AC *in vivo*, several reports performed mice experiments. A previous study performed oral administration of the ethanol extract of AC in SD rat, only antrodin B and C were detected in plasma (Liu et al., 2010). The metabolism and pharmacokinetics after oral administration of AC in male SD rats were studied (Qiao et al., 2015), and a total of 18 triterpenoids and 8 metabolites were detected in rat plasma after oral administration. Antcins K and H were the major exposure metabolites of AC. While the lanostanes were retained in the plasma at a low concentration for a considerable amount of time, the ergostanes were typically quickly absorbed and removed. Actin H was found in the tumor tissue of ICR mice with xenogra S180 tumor model after oral administration of AC extract (Li et al., 2018). In a Caco-2 cell monolayer model, most ergostanes exhibited high permeability (Wang et al., 2015). In detail, antcin H and antcin B could easily pass through the Caco-2 cell layer; antcins A, B, C, H, and K were absorbed through passive transcellular diffusion; and the permeability of lanostanes was poor, including dehydrosulphurenic acid, 15α-acetyldehydrosulphurenic acid, dehydroeburicoic acid, and eburicoic acid. Components could be absorbed in plasma is the basis for its entry into the brain regions by passing through the blood-brain barrier (BBB) or through the nose-to-brain pathway. The above-mentioned ergostanes in AC may exhibit biological activity for the treatment of neurologic diseases.

Using a SD rat model, the pharmacokinetic properties of ergosterol were investigated (Zhao et al., 2011). After a single oral administration of ergosterol (100 mg/kg) to SD rats, two metabolites (ERG1 and ERG2) were identified in the plasma, urine, and fecal samples. The side-chain was oxidized with β-d-glucopyranoside structure. The peak concentration (Cmax) time was 8.00 ± 1.18 h. Approximately 62.5% of the administered ergosterol was excreted from the feces, while 3.2% was eliminated from the urine. It remains unclear whether ergosterol can transport and metabolism in the brain.

The pharmacokinetics of antroquinonol by administration were evaluated in patients with metastatic non-small-cell lung cancer in a clinical study (Liao et al., 2023). The mean elimination half-life ranged from 1.30 to 4.33 h, regardless of the treatment dose. Four metabolites of antroquinonol were identified by NMR spectroscopic analysis from the male Wistar rats’ urine following oral treatment (Chen et al., 2014). These results suggest that antroquinonol has good bioavailability, but the biological activity of four metabolites of antroquinonol was not determined.

The ability of drugs to cross the blood-brain barrier is critical for their neuroprotective function. After oral intake of antroquinonol in mice, no adverse effects were observed, and antroquinonol could penetrate through the blood-brain barrier (Chang et al., 2012). And adenosine from AC can enter brain and the brain efflux index was shown to be capable of reaching 90.1%±1.5% (Isakovic et al., 2004). More reports showed that the extracts and some active metabolites from AC have neuroprotective function, but the ability of them to cross the blood-brain barrier were not be detected. Further investigations need to be performed to clarify the usable of the candidates for neurological diseases.

## 4 Neuroprotection potential of AC

As a rich source of biologically active metabolites, AC exerts excellent effects on a variety of physiological processes and produces various bioactivities. Many other utilizable activities of AC await discovery. Hence, AC has enormous potential for the development of new drugs. The most recent findings on the therapeutic benefits, underlying mechanisms, and active metabolites of AC in the management and avoidance of neurological disorders are thoroughly reviewed in this article.

### 4.1 Neuroprotective activities of AC extracts

There is increasing evidence that AC is an encouraging candidate for various neurological disorders (Wang et al., 2019). Here, we have summarized the effects of AC in chronic neurodegenerative diseases including AD, PD, and other acute neurodegenerative diseases, mainly stroke.

#### 4.1.1 Alzheimer’s disease

Neuroinflammation, the production of free radicals in the brain, amyloidogenic processing, and the ensuing Aβ cascade-triggered neuronal dysfunction and death are the pathological hallmarks of AD and other forms of dementia, which are the potential targets for AD treatment (Simpson and Oliver, 2020). Pharmacological therapy for neurodegenerative diseases provides only temporary symptomatic relief; hence, more effective drugs need to be developed (Passeri et al., 2022). Natural products with diverse structure and excellent activity are the main sources of new drugs.

The accumulation of Aβ in the brain may directly contribute to the degeneration of neurons during the pathogenesis of AD. Several isoforms of Aβ peptide have been isolated *in vivo* including Aβ1–38, Aβ1–39, Aβ1–40, Aβ1–42, and Aβ1–43. Aβ1–40 is the predominant sequence isolated from cerebrospinal fluid, while Aβ1–42 is the predominant component of senile plaques in parenchyma (Miyashita et al., 2009). Meanwhile, Aβ1-40, which is more abundantly produced by the cells than Aβ1-42, is commonly colocalized with Aβ1-42 in the plaque. Likewise, Aβ25-35 fragment can also induce aggregation and toxicity, similar to Aβ1-42. Aβ peptide is with important applications in the establishment of AD cell model. Methanol extract (10–50 μg/mL) from the wild fruiting body could suppress the inflammation induced by Aβ25-35 in EOC13.31 microglia at a dose-dependent manner, indicating that AC might be useful for the prevention of inflammation in the neurodegenerative brain (Liu et al., 2007). A study was conducted to evaluate the effect of AC fruiting body and mycelium on alleviating neurotoxicity induced by Aβ1-40 in the PC12 cell model and AD animal model (Wang et al., 2012). The results showed that AC can improve the memory and learning abilities by inhibiting several AD risk factors, including reactive oxygen species (ROS), p-tau, and BACE expression, as well as Aβ1-40 accumulation, suggesting that the fruiting body has stronger anti-oxidant and anti-inflammatory abilities to inhibit Aβ1-40-induced neurotoxicity than the mycelium. Another study also reported that AC ethanol extract (ACEE) protected PC12 cells activated by Aβ25-35 via increasing the BcL-2/Bax ratio to resist apoptosis, reducing ROS, and modulating adenosine A1 receptor (ADORA1) to enhance neuroprotective bioactivity and adenosine A2 receptor (ADORA2) to inhibit neurodegeneration. It also prevented the formation of Aβ25-35 fibrils and the production of tumor necrosis factor-alpha (TNF-α), ROS, malondialdehyde (MDA), and NO. Binding the function and the composition determination, it is demonstrated that the high content of triterpenoids, phenolics, and adenosine in ACEE was responsible for the rescue of these detrimental effects (Chang et al., 2012).

#### 4.1.2 Parkinson’s disease

Typically, PD is mainly caused by intra-cytoplasmic α-synuclein aggregation in the dopaminergic neurons of the substantia nigra compacta, leading to a decrease in the release of dopamine in the striatum. Several hypotheses of the dopaminergic cell death include mitochondrial dysfunction, iron accumulation, and inflammation (Morris et al., 2024). The preference for natural products is attributed to their efficiency and comparably fewer side effects.

1-Methyl-4-phenyl-1,2,3,6-tetrahydropyridine (MPTP) is one of the most used neurotoxins in PD animal models. Recently, an *in vivo* study in a MPTP induced PD mouse model demonstrated the protective role of AC extract administration significantly reduce α-synuclein-positive neuron numbers, and protect the brain from MPTP-induced loss of TH^+^ neurons, neuroinflammation, and oxidative stress to prevent dopaminergic cell death and glial activation (Lanza et al., 2023). 6-Hydroxydopamine (6-OHDA) is the most sought-after neurotoxin to identify oxidative stress-induced PD model *in vitro and in vivo* because it generates ROS and mimics the neuropathological and biochemical features, which can cause neuronal degradation and apoptosis in the dopaminergic neurons (Hernandez-Baltazar et al., 2017). Among solid-state-cultured mycelium of AC extracts, 11 metabolites belonging to quinone, phenolic acid derivatives, ubiquinone derivatives, alkaloids, and triterpenoid were identified to exhibit potent protective effects against 6-OHDA-induced toxicity to decrease dopaminergic neuronal loss in PC12 cells. The underlying mechanism likely involved the restoration of morphological changes in the nuclei, the reduction of ROS production and caspase 3 activity (Zou et al., 2022).

#### 4.1.3 Stroke

The most prevalent type of stroke is ischemic stroke, which causes degeneration and death of neurons. The pathology of ischemic stroke is extremely complex, oxidative stress and inflammation are the two major players (Chamorro et al., 2021). Neuroprotection is a promising strategy for stroke treatment. Natural products are reported to have excellent antioxidant and anti-inflammatory activities (Chen et al., 2020).

The activation of JNK and p38 and concurrent inhibition of ERK are critical for induction of apoptosis in both neuronal and non-neuronal cells (Xia et al., 1995). It has been reported that AC could effectively prevent serum-deprived apoptosis of PC12 cells by increasing phosphorylated ERK and decreasing phosphorylated JNK and p38, and by regulating a PKA/CREB-dependent pathway (Huang et al., 2005; Lu et al., 2008). Oral treatment of AC extract provided neuroprotection in rats with thromboembolic stroke by reducing infarct volume, improves neurological outcome (Lee et al., 2014). Furthermore, downregulation of iNOS/HO-1/Bax/caspase-3 and inhibition of hydroxyl radical formation were the main molecular pathways behind the AC neuroprotective activity against cerebral ischemia in rats (Yang et al., 2015). Ethyl acetate crude extract of AC (EtOAc-AC) showed protective effects both in acute ischemic stroke (AIS) injured mice and oxygen-glucose deprivation (OGD) induced Neuro 2A cells by inhibiting inflammation and apoptosis (Wang et al., 2019). Cobalt chloride (CoCl_2_) acts as hypoxia mimetic agent that increases the ROS production, resulting to the increase of inflammatory mediators such as TNF-α, interleukin 1 beta (IL-1β), inducible nitric oxide synthase (iNOS), and cyclooxygenase-2 (COX-2) (Zhong et al., 2014). In a recent study, the authors found that AC alcohol extracts (AC-AE) reduced cell damage against CoCl_2_-induced hypoxic toxicity in both C6 neuronal and C6 glial cells, and significantly reduced the stroke infarct size and decreased the level of proinflammatory iNOS and COX-2, and increased anti-inflammatory Nrf2 and HO-1 content in experimental rats, suggesting that AC-AE possesses protective effects in the ischemic stroke model (Kong et al., 2021).

Evidence from the above studies support that the medical benefits of AC can alleviate neuronal disorders. This great potential on the neuroprotective drug development of AC has further encouraged researchers to identify its functional metabolites.

### 4.2 Neuroprotective activities of isolated metabolites

The protective role of AC on neuronal disorders has been demonstrated using *in vitro* and *in vivo* experiments. Common pathways including anti-apoptosis, anti-oxidant, and anti-inflammatory play crucial roles in the degeneration and loss of axons and neurons (Moujalled et al., 2021). The effects of metabolites isolated from AC on neurological disorders are reviewed below.

#### 4.2.1 Anti-apoptotic activities

Extensive neuronal loss was observed in neuronal disorders, and the apoptosis marker activated caspase-3 has been observed in AD, PD, and stroke, which suggested that apoptosis plays crucial roles in the pathological processes (Robertson et al., 2000). A common feature of most neurological diseases is the degeneration of neurons, thus, drugs that inhibit neuronal apoptosis could thus be candidates for therapy of neurodegenerative disorders (D'Mello and Chin, 2005). ADORA2 encodes the G protein-coupled adenosine receptor known as adenosine receptor subtype A2A, which can promote neuronal polarization and axon formation (Alçada-Morais et al., 2021). It is reported that adenosine (ADO) from AC could protect PC12 cells from serum deprivation by activation of the ADORA2 (Lu et al., 2006). Further research showed that adenosine suppressed JNK and p38 activities through a protein kinase A (PKA) pathway in serum-deprived PC12 cells (Lu et al., 2008).

#### 4.2.2 Antioxidant activities

Increasing ROS is demonstrated to be susceptible to neuronal damage and functional deficits, which results in neurological disorders including AD, PD, and ischemic stroke (Teleanu et al., 2022). Brain tissue is characterized by high levels of oxygen consumption and high metabolic demand, but relatively low levels of antioxidant enzymes (HO-1, SOD, CAT, and GPx) and non-enzymatic antioxidants (vitamin A, C, and E) (Halliwell, 1992). Therefore, supplementation of antioxidants represents an effective strategy of prevention and treatment to restore the functionality and survival of neuronal cells against oxidative stress.

Current evidence has shown that AC is a potent scavenger of direct oxygen free radicals that protects cells from oxidative damage. Polysaccharides isolated from AC (ACP) have been found to comprehensively improve the neuroethology of PD mice, including autonomic activity, coordination, motility ability, and cognitive ability. Investigation of the mechanisms showed that ACP can enhance the expression levels of dopamine and dihydroxyphenylacetic acid in the striatum, and significantly decrease the expression of NLRP3 inflammasome and downstream inflammatory factors in a dose-dependent manner (Han et al., 2019). NLRP3 inflammasome can be activated not only by a variety of exogenous pathogens, but also by certain endogenous signals and metabolites. ROS is an upstream signal for NLRP3 activation (Hernandes et al., 2014). Further study demonstrated that ACP intervention can lead to significantly decreased ROS-NLRP3 activation, reduce intracellular ROS, and the apoptotic rate at the cellular and *in vivo* levels (Han et al., 2020).

A recent study in amyloid precursor protein (APP) transgenic mice revealed that antroquinonol, a ubiquinone derivative isolated from AC, might significantly improve memory acquisition, alleviate Aβ plaque pathology, and inflammation, mainly by decreasing histone deacetylase 2 (HDAC2) and increasing Nrf2 levels (Chang et al., 2005). As the catalytic subunit of deacetylase repressor complexes, the increase of HDAC2 level and activity in AD have been linked to the worsening of neuronal and synaptic function (Gonzalez-Zuninga et al., 2014). Further antroquinonol administration studies showed that anxiety-related behavior and cognitive abilities in 3XTgAD mice could be significantly improved; furthermore, inflammatory markers, AD biomarkers, and oxidative stress markers showed a significant decrease (Francesca et al., 2022). The results are consistent with previously study in the APP transgenic mice, which confirmed the antioxidant capacity of antroquinonol in AD model.

#### 4.2.3 Anti-inflammatory activities

The prominent role inflammation plays in various age-related diseases such as AD, PD, and central nervous system (CNS) injury (Khadka et al., 2020). Glial cells and immune cells are associated with the instigation of neuroinflammation. Plenty of potential inflammatory targets for intervention have been proposed (DiSabato et al., 2016).

Several intracellular signaling and transcription factors mediated by the activation of microglia in turn activate the inflammatory pathway in cerebral haemorrhage. The US patent US20180353520 has claimed that the active metabolites dehydroeburicoic acid, dehydrosulphurenic acid, and 4,7-dimethoxy-5-methyl-1,3-benzodioxole from AC can be used for the treatment of stroke (Wu et al., 2018). Their anti-inflammatory bioactivities have been demonstrated by several studies (Shen et al., 2004; Deng et al., 2013; Shie et al., 2016). Oxidative stress is reported to play an important role in the activation of the inflammatory cascade (Fan et al., 2023). Antcin C is a well-known metabolite of AC, which exerts its hepatoprotective and antioxidative activities by modulating the Nrf2 pathway. It is also reported that antcin C treatment in rats with cerebral injury could reduce the oxidative stress parameters and inflammatory cytokine levels via inhibiting the TLR-4 pathway (Ling et al., 2020).

Ergosta-7,9 (11),22-trien-3β-ol (Ergostatrien-3β-ol; EK100), a triterpenoid metabolite abundant in both the fruiting bodies and (Chao et al., 2021), also showed promising medicinal uses in neurological disorders. EK100 and antrodin C effectively improved the autonomous behavior and social ability and alleviated amyloid plaque burden in APP/PS1 mice (Tsay et al., 2021). EK100 from AC improved AD symptoms in a *Drosophila* model with Aβ42 overexpression by preventing the activation of microglial (Liu et al., 2021). EK100 showed a significant anti-inflammatory effect against LPS-induced NO production in BV2 cells, with IC50 values of 18 ± 2 μM, which was almost as potent as the NF-κB inhibitor. In the AIS mouse model, EK100 treatment reduced ischemic brain injury by decreasing the expression of p65 and caspase 3. EK100 also promoted endogenous neurogenesis through GSK-3 inhibition and β-catenin activation by activating PI3K/Akt signaling, suggesting EK100 may have other targets in addition to anti-inflammatory activity and it may act as potent brain protective agent (Wang et al., 2019). In another study, EK100 treatment showed neuroprotective effects on ipsilateral injuries in a mouse model of collagenase-induced intracerebral hemorrhage (ICH) via inhibiting COX-2 and MMP-9; EK100 treatment also exerted significant anti-inflammatory function by downregulating JNK activation in BV-2 cells and ICH mice (Hsueh et al., 2021; Huang et al., 2021). The above reports demonstrated that EK100 isolated from AC showed obvious anti-inflammatory effects through interfering with several pathways and anti-neuropathy *in vivo* and *in vitro*.

Our previous study showed that ergosterol isolated from AC, an isomer of EK100, could suppress the activation of BV2 treated with LPS by inhibiting the NF-κB, MAPK, and AKT signaling pathways (Sun et al., 2023). Further investigation in LPS-treated ICR mice found that ergosterol exhibited anti-neuroinflammatory activity and maintained the synaptic proteins. Another group also reported that ergosterol isolated from *Auricularia polytricha* or *Cordyceps militaris* attenuated neuroinflammation in BV2 cells (Nallathamby et al., 2015; Sillapachaiyaporn et al., 2022a), and exhibited neuroprotective activity in TNF-α-induced HT-22 cells via regulating the expression of N-methyl-D-aspartate receptors (NMDARs) and antioxidant enzyme, and cell survival pathways (Sillapachaiyaporn et al., 2022b). Therefore, ergosterol exerts its neuroprotective effect mainly through its anti-neuroinflammatory property via multiple pathways.

#### 4.2.4 Stimulation of neurite regrowth and NGF synthesis

Neurite regrowth promoters were found to be possible therapies for neurological disorders. These neuritogenic substances have the ability to stimulate the growth of neurites in neuronal cells and are expected to be effective in the treatment of nerve injuries (More et al., 2012). Nerve growth factor (NGF), brain derived neurotrophic factor (BDNF), neurotrophin 3 (NT-3), and glia-derived neurotrophic factor (GDNF) are identified as important factors called neurotrophins for the survival and differentiation of neurons as well as neuronal maintenance (Numakawa and Kajihara, 2023). Neurotrophic factors are one of the key mediators of neural plasticity and functional recovery. They have great potential as therapeutic drugs against neurodegenerative diseases.

Some lanostane triterpenoids such as tumulosic acid, polyporenic acid C, 16α-hydroxyeburicoic acid, dehydrotumulosic acid, and pachymic acid are also widely found in medicinal mushrooms other than AC and can induce neurite outgrowth in human astrocytoma 1321N1 and PC12 cells, which might be due to inducing the expression of neurotrophic factors, NGF, and BDNF (Hassan et al., 2022). Further investigation demonstrated that these metabolisms identified as neurotrophins show weak cytotoxic effects on mammalian cells, but they are lacking certain structural features. It is worthwhile to further study these fungi, including their secondary metabolisms, the function structural and so on, which might reveal the interesting chemotaxonomic relationships as well as hitherto unprecedented biological activities of the constituents from AC.

#### 4.2.5 Inhibition of beta-amyloid toxicity

Aβ peptide is formed by a cleavage process of APP via beta-cleavage by the secretases beta (BACE1) and gamma-cleavage of gamma secretases. Oligomeric and fibrillar beta-amyloid are both toxic to neurons. Aβ-dependent neuronal death can cause changes in cell membrane fluidity and integrity, leading to ion leakage, disruption of cellular calcium balance, decreased membrane potential, ultimately promoting apoptosis and synaptic loss (Silva et al., 2023).

Five metabolites—19-hydroxylabda-8 (17)-en-16, 15- olide; 3b,19-dihydroxylabda-8 (17), 11E-dien-16,15-olide; 13-epi-3b, 19-dihydroxylabda-8 (17), 11E-dien-16, 15- olide; 19-hydroxylabda-8 (17), 13-dien-16,15-olide, and 14-deoxy-11, 12-didehydroandrographolide—were obtained from the fruiting bodies of AC, and showed neuronal protection against Aβ25–35 damage (Chen et al., 2006). The above-mentioned studies have been demonstrated to be the basic substance of AC for neuroprotection.

Metabolites derived from AC possess potential neuroprotective properties via its various bioactivities. We have summarized the effects of some active metabolites from the AC on CNS diseases in Table 1.

**TABLE 1:** Summary of the main *in vitro* and preclinical studies on *Antrodia camphorata* and some active metabolites in the CNS diseases.

Extract/metabolite	Model	Main effects	References
Water and ethanol extract of mycelium and fruiting body	Aβ1-40-induced PC12 cells and Aβ1-40 infusion AD rats	Improved working memory ability	Wang et al. (2012)
Ethanolic extract	Aβ_25–35_ induced PC12 cells	Rescued neuronal death by suppressing ROS and modulated ADORA1 and ADORA2A	Chang et al. (2012)
Methanol extract of fruiting body	Aβ_25–35_-induced EOC13.31 cells	Inhibited both iNOS and cyclooxygenase-2 (COX-2) expression	Liu et al. (2007)
Commercial AC extract	MPTP-induced PD model mice	Increased tyrosine hydroxylase expression, reduced alpha-synuclein-positive neurons number, attenuated the neuroinflammatory state, reduced oxidative stress	Lanza et al. (2023)
Solid-state-cultured mycelium	6-OHDA-induced PC12 cells	Increased cell viability, inhibited cell apoptosis, upregulated tyrosine hydroxylase and dopamine transporter levels, downregulated α-synuclein level	Zou et al. (2022)
Ethanolic mycelial extract	Serum deprivation-induced PC12	Prevented cell apoptosis through MAPK and PKA/CREB pathway	Huang et al. (2005)
			Lu et al. (2008)
Commercial crude extracts	MCAO-induced ischemia rats	Reestablished blood flow, inhibited HO-1, inflammatory responses apoptosis, and free radical formation	Yang et al. (2015)
			Kong et al. (2021)
Alcohol extracts	CoCl_2_-induced C6/PC12 cells and stroke rat	Reduced cell damage and decreased ROS production, stroke infarct size and MDA level and increased the level of antioxidants	Lee et al. (2014)
Ethyl acetate crude extract	Mice AIS model	Activated PI3K/Akt and inhibited GSK-3 to decrease p65NF-κB, caspase 3 and promote neurogenesis	Wang et al. (2019b)
ACP	MES23.5 cells and 6-OHDA-induced PD mice	Improved the neurobehavioral behavior, inhibited ROS-NLRP3 signaling	Han et al. (2019)
			Han et al. (2020)
Antcin C	Cerebral injured rats	Reduced the neurological scores and volumes, oxidative stress and cytokine levels; ameliorated TLR-4, IRAK4, and zonula occludens-1 proteins	Ling et al. (2020)
Antrodin C	APPswe/PS1dE9 mice	Reduced amyloid deposits and promoted nesting behavior through microglia and perivascular clearance	Tsay et al. (2021)
EK100	Mice AIS model	Activated PI3K/Akt and inhibited GSK-3 to decrease p65NF-κB, caspase 3 and promote neurogenesis	Wang et al. (2019a); Hsueh et al. (2021); Huang et al. (2021); Liu et al. (2021)
	Mice ICH model	Inhibited microglial JNK activation, attenuated brain COX-2 expression, MMP-9 activation, inflammatory cytokines and brain injuries	
	AD model	Reduced microglia activation, decreased caspase 3 expression; improved the life span, motor function, learning, and memory	
Ergosterol	LPS- or BPA-induced BV2 cells	Inhibited the NF-κB, AKT, and MAPK signaling pathways and pro-inflammatory cytokine levels, restored synaptic proteins expression	Nallathamby et al. (2015); Sillapachaiyaporn et al. (2022a); Sillapachaiyaporn et al. (2022b); Sun et al. (2023)
	LPS-induced ICR mice		
	TNF-α-induced HT22 cells	Promoted neuronal survival via Akt/GSK-3β signaling	
Antroquinonol	APPswe/PS1dE9 mice	Improved learning and memory, reduced hippocampal Aβ levels and astrogliosis, increased Nrf2 and decreased histone deacetylase 2 levels	Chang et al. (2005)
	3XTgAD mice	Improved behavior and cognitive abilities, reduced systemic inflammatory markers, AD biomarker levels, oxidative markers	Francesca et al. (2022)
Adenosine	Serum deprivation-induced PC12	Activated A (2A)-R to prevent cell apoptosis	Lu et al. (2006); Lu et al. (2008)
19-hydroxylabda-8 (17)-en-16,15-olide	Aβ_25-35_-treated Cortical neurons	Protected neuronal viability	Chen et al. (2006)
3â, 19-dihydroxylabda-8 (17), 11E-dien-16,15-olide			
13-epi-3â,19-dihydroxylabda-8 (17), 11E-dien-16,15-olide			
19-hydroxylabda-8 (17), 13-dien-16,15-olide			
14-deoxy-11,12-didehydroandrographolide			
Tumulosic acid polyporenic acid C 16α-hydroxyeburiconic acid	Rat 1321N1 cells and PC12 cells	Stimulated neurite outgrowth, increased NGF and BDNF levels	Hassan et al. (2022)

## 5 Potential with the gut-microbiome-brain axis of AC

The gut microbiome plays a key role in human health, such as overall homeostasis maintenance, immune system moderation, and central nervous system regulation. Increasing clinical and preclinical evidence points to the existence of the microbiota-gut-brain axis that forms a bidirectional network between the CNS and the gut (Singh et al., 2016; Sharon et al., 2019). Microbial imbalance is particularly linked to various neurological disorders including AD (Cattaneo et al., 2017), PD (Forsyth et al., 2011), and stroke (Sorboni et al., 2022). Plenty of research has attempted to define the molecular cross-talk between the host and microbiome to provide novel perspectives on promising therapeutic approaches for the management of neurological disorders (Mou et al., 2022).

A previous study in the leptin-induced Caco-2 cells model found that AC had a positive effect on intestinal microflora by repairing intestinal-barrier damage and enhancing the integrity of the intestinal barrier (Tsai et al., 2020). Another study in high-fat diet (HFD)-fed mice demonstrated that AC showed protective effects, maintained the intestinal barrier integrity, reduced the Firmicutes/Bacteroidetes ratio and increased *Akkermansia muciniphila* level (Chang et al., 2018). It is also reported that solid-state cultured AC can reduce hyperglycemia and tend to alleviate metabolic disorder in HFD-induced obese mice, mainly reduce the relative abundance of Firmicutes-to-Bacteroidetes ratio and elevate the relative abundance of *Akkermansia* spp. (Wang et al., 2020). The arrangement of the enterocyte was not disrupted by AC extract treatment, but intactness and denseness of hepatic tissue was elevated by regulating redox and cytoskeleton-related proteins and increasing the abundance of *Akkermansia* spp. in the gut microbiota of C57BL/6 mice (Tsai et al., 2021). *Akkermansia* spp. has reported to improve the situation of AD and PD (He et al., 2022). The above studies demonstrated that AC has neuroprotective potential in the gut microbiome by increasing probiotic bacteria *Akkermansia* spp.

In addition to crude extracts, the functions of AC metabolites on the microbiota-gut-brain axis have also been studied. In LPS-stressed slow-growing broiler breeds model, dietary supplement with 100–400 mg/kg ACP showed beneficial effects on liver damage and the bacterial microbiota species richness and diversity (Ye et al., 2022). Additionally, ACP inhibited the rise of Proteobacteria in LPS-induced group, while restored the beneficial cecal microbiota (typically *Lactobacillus*, *Faecalibacterium*, and Christensenellaceae R-7 group). Thus, ACP enhanced the species richness, and diversity indices might be related to the anti-inflammatory effect. Intragastric administration of exopolysaccharides from AC in lincomycin hydrochloride (LIH)-induced mice greatly reduced serum inflammatory cytokine levels and alleviated immune organs damage, while also regulating the microbial environment via enhancing the relative abundance of beneficial microbiota in the intestine (typically *Lactobacillus*, *Roseburia*, *Ligilactobacillus*, and Lachnospiraceae_NK4A136_group), and reducing the relative abundances of harmful microbes such as *Enterococcus* and *Shigella* (Lu et al., 2022), which were increased in AD subjects (Hou et al., 2021). Antrodin A from AC can alleviate the alcohol-induced metabolic disorders via regulating the composition of intestinal microbiota by decreasing *Clostridium sensu stricto 1*, Lachnospiraceae_NK4A136_group, Prevotellaceae_NK3B31_group, and Prevotellaceae_UCG-001, and increasing the relative abundance of *Lactobacillus* and *Dubosiella* (Yi et al., 2021). Another study demonstrated that the ethyl acetate layer of AC mycelium extract, especially antrodin A, and antroquinonol decreased the abundance of intestinal Helicobacteraceae and increased the relative abundance of Lachnospiraceae and Ruminococcaceae to prevent alcohol-induced oxidative stress and inflammation in mice (Yi et al., 2020). The decrease of Ruminococcaceae could act as a predictive marker for the rapidly progressive mild cognitive impairment (Yang et al., 2023). According to these studies, AC metabolites may have the potential to modulate the brain-gut axis.

**FIGURE 2:**
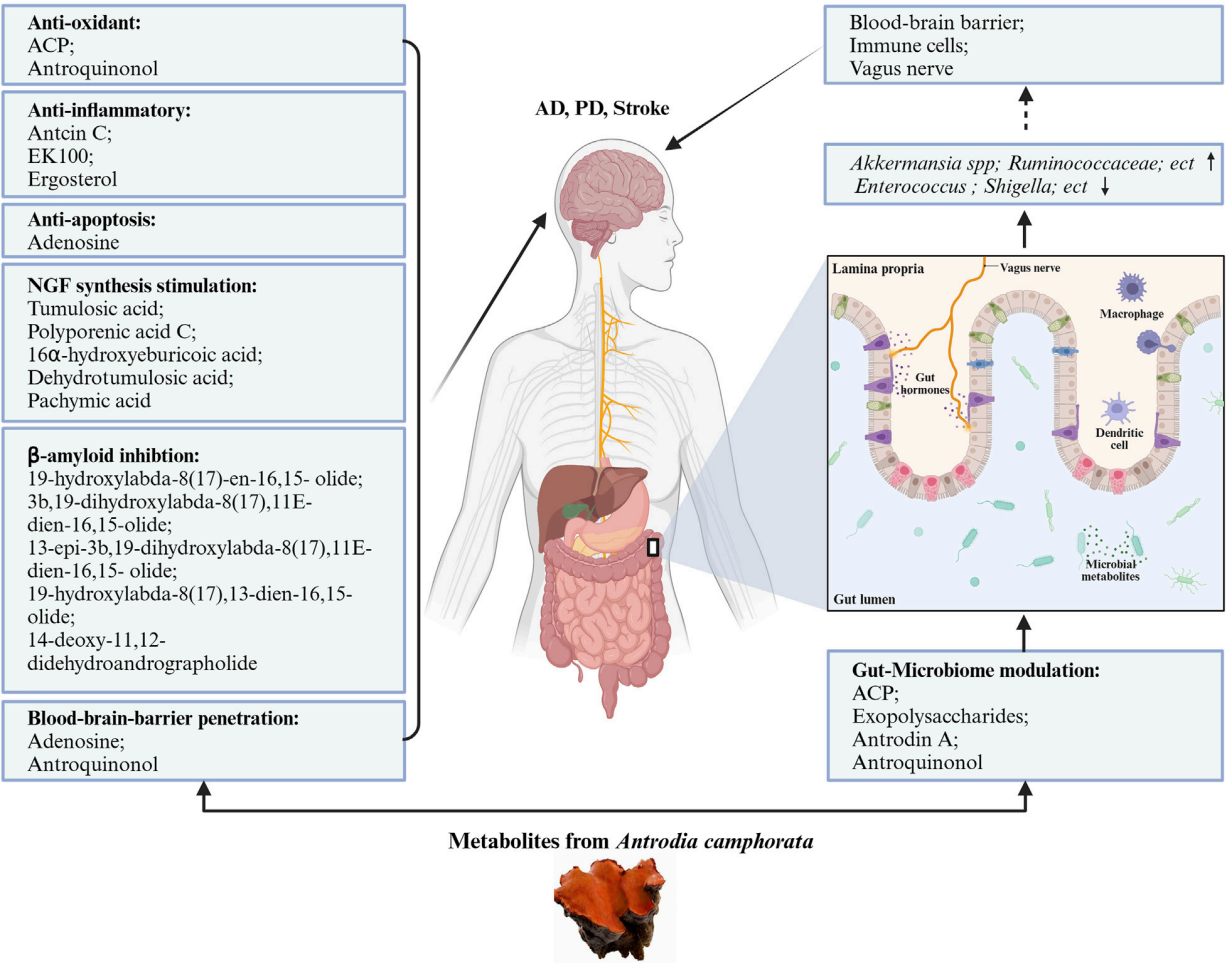
Neuroprotective potentials of *Antrodia camphorata.* The bioactive metabolites from AC play neuroprotective function directly by anti-oxidant, anti-inflammatory, anti-apoptotic, stimulation NGF synthesis, and beta-amyloid inhibition pattern, also indirectly by gut-microbiome modulation.

The composition of the gut microbiota is important for host homeostatic functions. AC and its active metabolites showed prebiotic effects on the gut microbiome to affect the state of the brain and nervous system. AC might show its neuroprotective activity on the gut-microbiota-brain axis. We summarized the effects of AC in the gut microbiome in Table 2. Figure 2 presents the neuroprotective effects of bioactive molecules contained within AC by working on nervous system and gut-microbiota.

**TABLE 2:** Summary of the effects of *Antrodia camphorata* treatment on the GM in different models.

Model	Treatment	Increased bacteria	Decreased bacteria	References
HFD-induced mice	Ethanol extract; 230 mg/kg/day by gavage for 14 weeks	*Akkermansia muciniphila*	*Mucispirillum* and *Blautia*	Chang et al. (2018)
	Water extract	*Streptococcus* spp., *Eubacterium* spp., *Eggerthella lenta*, *Clostridium methylpentosum*, *A. muciniphila*	Ruminococcaceae and Lachnospiraceae at the family level	Wang et al. (2020)
	0.009 and 0.09 g/kg/day for 8 weeks		*Clostridium scindens* and *Clostridium cocleatum* at the species level	
C57BL/6 mice	Solid-state cultivated mycelium; 1.6667 g/kg for 4 weeks	*Alistipes shahii*		Tsai et al. (2021)
ICR mice	Exopolysaccharides	*Lactobacillus*, *Roseburia*, *Ligilactobacillus*, and Lachnospiraceae_NK4A136_group	*Enterococcus* and *Shigella*	Lu et al. (2022)
	0.08, 0.25, 0.75 g/kg 12 days			
LPS-induced chicken	ACP; 100, 200, and 400 mg/kg for 21 days	Proteobacteria	Firmicutes, *Lactobacillus*, and *Synergistes*	Ye et al. (2022)
		*Alistipes*, *Ruminiclostridium*, *Megamonas*, [*Ruminococcus*] torques group and *Campylobacter*		
Alcoholic liver injury mice	Antrodin A	Firmicutes, *Lactobacillus,* and *Dubosiella*	*Bacteroidetes*, Lachnospiraceae_NK4A136_group*, Prevotellaceae_NK3B31_ group, Prevotellaceae_UCG-001, and Clostridium_ sensu_stricto_1*	Yi et al. (2020); Yi et al. (2021)
	75, 150 mg/kg for 15 days			
	Antrodin A; 100, 200 mg/kg/day for 12 days	Muribaculaceae*, Lactobacillus,* Lachnospiraceae	Erysipelotrichaceae*,* Helicobacteraceae*,* Proteobacteria	
		Firmicutes, Bacteroidetes		
		Ruminococcaceae		

## 6 Future perspectives

AC is a valuable and medicinal fungus known widely and has tremendous medicinal properties and nutritional value for brain health. It possesses several health-endorsing properties such as anti-oxidant, anti-apoptosis, and anti-inflammatory and probably improves the gut microbiome in neurologic disorders. In general, the isolated metabolites from AC have not been deeply and systematically studied in neuroscience. There are still metabolites that have not been identified; hence, AC has great potential. For metabolites that have entered clinical evaluation, production should be increased by applying different strategies. Furthermore, in-depth pharmacokinetic studies and modification of AC metabolites are also lacking. The following aspects of AC research could be improved:

### 6.1 Increase the production of the bioactive metabolites in AC

Genomic and transcriptomic analyzes will help to develop strategies to increase the production of useful metabolites (Lu et al., 2014). For tissue-specific metabolites, secondary metabolites biosynthesis pathway genes were enriched, including 14-α-demethylase (CYP51F1) for the conversion of lanosterol to ergosteroidal triterpenes in fruiting bodies, coenzyme Q (COQ) for the synthesis of antroquinonol in mycelia, and polyketide synthase for the synthesis of antrocamphin in fruiting bodies. It was reported that talc enhanced the yield of the bioactive secondary metabolite antioxidant antrodin C in the submerged fermentation of AC by increasing the permeability and fluidity of the cell membrane, upregulating the key genes and then improving the biosynthesis process (Fan J. H. et al., 2023). Another study showed that oxidative stressors supplementation (such as hydrogen peroxide) can increase the yield of antrodin C in submerged fermentation of AC (Hu et al., 2020). For instance, biosynthesis is a strategy to achieve industrial-scale production for the identification of novel metabolites and improvement of the yield of previously known valuable metabolites.

### 6.2 Specific investigation of metabolite pharmacology and pharmacokinetics

As an emerging medicinal fungus, AC has a variety of important biological activities and pharmacological functions, a variety of bioactive metabolites from its fruiting bodies and mycelia that have been isolated and purified, but the underlying mechanism such as the pharmacokinetics and pharmacodynamics remain unclear, which are important for further biochemical research and clinical trials. The establishment of an intracerebral pharmacokinetic research model will truly reflect the process of treatment and action of drugs after entering the brain tissue, ensuring the effective concentration of brain-targeted drugs in the brain and preventing the damage of non-brain-targeted drugs to neurological function, thereby greatly improving the effectiveness and safety. Most pharmacological studies were derived from *in vitro* laboratory investigations; hence, further preclinical studies are necessitated for a full evaluation of these valuable metabolites from AC *in vivo* experiments to promote clinical applications and complete the clinical validation for potential therapeutic benefits.

### 6.3 Strategies for bioavailability improvement of the metabolites from AC

Structure function studies are important for the design of more effective drugs, and many pharmacological actions and its molecular mechanisms are defined. The structures of terpenoids isolated from AC are closely related to the anti-inflammatory activity. The substituents on C15 are crucial to the anti-inflammatory properties of lanostane-type metabolites (Yang et al., 2022).

Chemical modification is necessary to enhance the structural diversity and druggability of natural products. It has been demonstrated that side-chain esterification increased the antitumor activity of *Antrodia* ergosteroids (Li et al., 2020). Furthermore, a number of investigations have shown that amide derivatives of triterpenoids containing nitrogen heterocycles or anilines at the side chain exhibit higher cytotoxicity than the original metabolites (Li et al., 2021). Ergosterol lacks aqueous solubility, but a study found that solubility and bioavailability can be improved by using nanostructured lipid carriers (NLCs). Nanoparticles drug delivery system can also improve the bioavailability of ergosterol following oral administration (Zhang et al., 2016). AC modified the nanocarrier β-cyclodextrin (BCD) inclusion complex could potentially unlock its full potential and attempt to make it suitable for improving skeletal muscle health and possible to develop evidence-based drug (Menon et al., 2022). The blood-brain barrier penetration of potential drugs is a critical issue, and delivery of these neuroprotective metabolites into the brain requires a tremendous effort, for which nanomedicine has emerged as a promising approach.

## Figures and Tables

**Table:** 

**6-OHDA**	6-Hydroxydopamine
**Aβ**	amyloid-β
**AC**	Antrodia camphorate
**ACEE**	Ethanol extract of AC
**ACP**	polysaccharides isolated from AC
**AD**	Alzheimer’s disease
**ADO**	adenosine
**ADORA1**	adenosine A1 receptor
**ADORA2**	adenosine A2 receptor
**AIS**	acute ischemic stroke
**APP**	amyloid precursor protein
**BACE1**	beta-cleavage by the secretases beta
**BCD**	β-cyclodextrin
**BDNF**	brain derived neurotrophic factor
**Cmax**	peak concentration
**CNS**	central nervous system
**CoCl2**	Cobalt chloride
**COQ**	coenzyme Q
**COX-2**	cyclooxygenase-2
**CYP51F1**	14-α-demethylase
**EFSA**	European Food Safety Authority
**EK100**	Ergosta-7,9 (11),22-trien-3β-ol
**EtOAc-AC**	Ethyl acetate crude extract of AC
**FDA**	Food and Drug Administration
**GDNF**	glia-derived neurotrophic factor
**HFD**	high-fat diet
**HIF-1a**	hypoxia-inducible transcription factor-1a
**ICH**	intracerebral hemorrhage
**IL-1β**	interleukin 1 beta
**iNOS**	inducible nitric oxide synthase
**LIH**	lincomycin hydrochloride
**MDA**	Malondialdehyde
**MPTP**	1-Methyl-4-phenyl-1,2,3,6-tetrahydropyridine
**NGF**	Nerve growth factor
**NLCs**	nanostructured lipid carriers
**NMDARs**	N-methyl-D-aspartate receptors
**NOAEL**	no-observed-adverse-effect-level
**NT-3**	neurotrophin 3
**OGD**	oxygen-glucose deprivation
**PD**	Parkinson’s disease
**PKA**	protein kinase A
**ROS**	Reactive oxygen species
**SD rats**	Sprague–Dawley rats
**TNF-α**	tumor necrosis factor-alpha
